# Nano-Medicines a Hope for Chagas Disease!

**DOI:** 10.3389/fmolb.2021.655435

**Published:** 2021-06-01

**Authors:** Satabdi Datta Choudhury

**Affiliations:** Department of Zoology, Shri Guru Granth Sahib World University, Fatehgarh Sahib, India

**Keywords:** Chagas disease, drug resistance, nanopharmaceuticals, nano-bio sensors, nano-vaccine

## Abstract

Chagas disease, is a vector-mediated tropical disease whose causative agent is a parasitic protozoan named *Trypanosoma cruzi.* It is a very severe health issue in South America and Mexico infecting millions of people every year. Protozoan *T. cruzi* gets transmitted to human through *Triatominae*, a subfamily of the Reduviidae, and do not have any effective treatment or preventative available. The lack of economic gains from this tropical parasitic infection, has always been the reason behind its negligence by researchers and drug manufacturers for many decades. Hence there is an enormous requirement for more efficient and novel strategies to reduce the fatality associated with these diseases. Even, available diagnosis protocols are outdated and inefficient and there is an urgent need for rapid high throughput diagnostics as well as management protocol. The current advancement of nanotechnology in the field of healthcare has generated hope for better management of many tropical diseases including Chagas disease. Nanoparticulate systems for drug delivery like poloxamer coated nanosuspension of benzimidazole have shown promising results in reducing toxicity, elevating efficacy and bioavailability of the active compound against the pathogen, by prolonging release, thereby increasing the therapeutic index. Moreover, nanoparticle-based drug delivery has shown promising results in inducing the host’s immune response against the pathogen with very few side effects. Besides, advances in diagnostic assays, such as nanosensors, aided in the accurate detection of the parasite. In this review, we provide an insight into the life cycle stages of the pathogen in both vertebrate host and the insect vector, along with an overview of the current therapy for Chagas disease and its limitations; nano carrier-based delivery systems for antichagasic agents, we also address the advancement of nano vaccines and nano-diagnostic techniques, for treatment of Chagas disease, majorly focusing on the novel perspectives in combating the disease.

## Introduction

The Chagas disease is also known as American Trypanosomiasis is a zoonotic disease caused by the protozoan parasite *T. cruzi* (Dutkiewicz-Serdynska, 1988; Franco et al., 2020; Hu et al., 2020). In addition to Latin America where nearly eight million people are affected by this infection (da Silveira et al., 2005), this disease is also rapidly spreading to other non-endemic countries, namely Australia, Canada, Japan, Europe (Requena-Mendez et al., 2015), and the United States of America (USA) (Echeverria and Morillo, 2019). The transmission of this infection mainly occurs by fecal contamination of *Triatominae*, a subfamily of the Reduviidae insects via insect bites or injured skin site (Andrade et al., 2014), the other ways of transmission of this parasite may also occur by organ transplantation, blood transfusion or from contaminated food (Ferreira et al., 2016; Hotez et al., 2019). The life cycle of the parasite requires two hosts: the invertebrate vector (triatomine insects) and vertebrate hosts (humans/animals), its life cycle consists of three stages epimastigotes, trypomastigote, and amastigote. The clinical symptoms of Chagas disease consist of two phases, and usually asymptomatic acute phase and a chronic phase that may be associated with digestive and cardiac lesions, that may eventually lead to cardiac failure (Perez-Molina and Molina, 2018b). However, neither the government in endemic countries nor the pharmaceutical companies have shown keen interest to elevate the quality of the available therapeutic options for the treatment of this disease (Prayag et al., 2020). Thus, in 1975, trypanosomiasis was classified as one of the neglected diseases by the World Health Organization (WHO) to attract the attention of the governmental and private agencies toward this disease and inspire them to develop more efficient therapies against this fatal infection (Ferreira et al., 2016). Till date, the nitro-heterocyclic compounds benznidazole and nifurtimox are the two major drugs approved by food and drug organization for the treatment of trypanosomiasis (Bern, 2011). But, long-term therapy, with these drugs is generally associated with serious side effects (Gutteridge, 1985; Castro and Diaz de Toranzo, 1988; Santamaria et al., 2014) and often be hindered by the problem of resistance development (Wilkinson et al., 2008; Campos et al., 2014). Much research has been conducted to date for the development of vaccines against Chagas disease (Sanchez-Valdez et al., 2015). However, the major difficulty in this process has been finding a protective antigen that will not produce any pathological effect in long run. Thus, the control measures for Chagas disease are still limited to diagnosis and treatment in absence of an ideal vaccine (Perez-Molina and Molina, 2018a).

Recently many nanocarriers have shown a promising role in increasing the efficacy and lowering down the toxicity of drugs, antigens, and adjuvant (Lynch and Cheng, 2007; Scire et al., 2019). Many recent reports also suggest that in comparison to conventional drugs and diagnostic strategies, the use of nanocarriers and nano-diagnostic strategies results in better management and treatment of the infection (Barratt, 2000; Hauck et al., 2010). The current review will provide an insight into the life cycle stages of the pathogen in both host and the insect vector, along with a brief overview of the current therapy for Chagas disease and its limitations; nano carrier-based delivery systems for antichagasic agents, and the advancement of nano-vaccines and nano-diagnostic techniques, for treatment of Chagas disease.

## Life Cycle and Pathogenicity of Chagas Disease Causative Agent

The causative agent of Chagas disease is a parasitic protozoan *T. cruzi*. The vector of this infection is a bug belonging to genera Triatoma and human as host (Prayag et al., 2020). In the early stage, the disease affects the circulatory and lymphatic systems, and in the late-stage, it spreads to the cardiac tissues and the tissues of the gastrointestinal tract (Matsuda et al., 2009; Teixeira et al., 2011; Pinazo et al., 2014a; Rodrigues-Dos-Santos et al., 2018).

**FIGURE 1:**
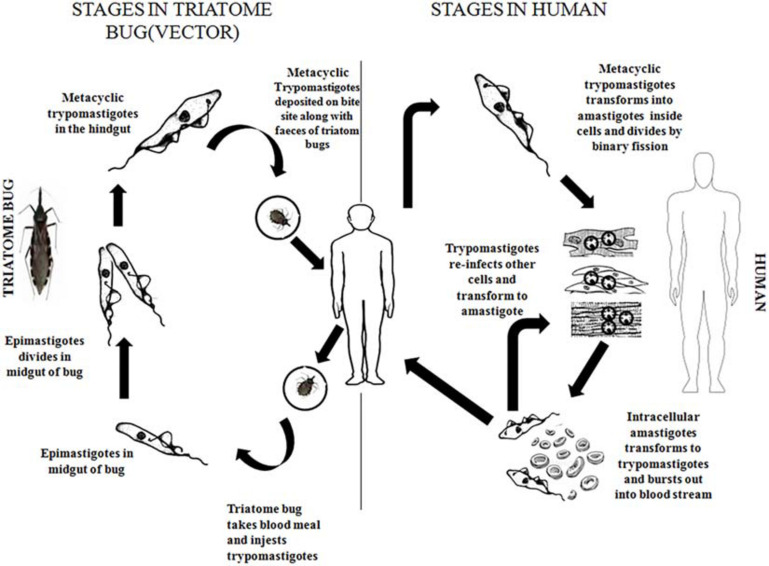
Life cycle of *Trypanosoma cruzi* in human and Triatome bug.

The infected vector deposits its feces carrying the parasite near the site of the bite wound, through this wound the parasite enters the bloodstream of the vertebrate host. The parasite exists in two forms namely metacyclic trypomastigote (infective form) and amastigote (replicative form) in the vertebrate hosts. The trypomastigote form causes intercellular infections, upon entering the cells the trypomastigote transforms to amastigote form where it divides by binary fission. Amastigotes then leave the infected tissues and are transformed into metacyclic trypomastigotes in the bloodstream (Prayag et al., 2020). In the insect vector, the parasite’s life cycle starts when the insect sucks the blood of the infected vertebrates bearing the trypomastigotes. Inside the stomach of the vector, the surviving trypomastigotes transform into the epimastigote stage a few days later (Castro et al., 2007). These epimastigotes migrate to the intestine, where they multiply and attach to the perimicrovillar membranes which are secreted by intestinal cells of the posterior midgut (Alves et al., 2007; Nogueira et al., 2007). In the perimicrovillar membranes, the non-infective epimastigotes transform into highly infective trypomastigotes known as metacyclic trypomastigotes which start the next phase of infection in the vertebrate hosts (Matthews, 2005; Terra and Ferreira, 2020) (Figure 1).

Metacyclic trypomastigote possesses a set of surface glycoproteins that enables the parasite to interact and invade the vertebrate host’s cells (Ramirez et al., 1993; Santori et al., 1996; Ruiz et al., 1998). The gp82 glycoprotein is one such glycoprotein that acts as an adhesion molecule and attaches with host cells in a receptor-mediated manner, triggering mobilization of Ca^2+^ ions (Dorta et al., 1995; Ferreira et al., 2014) and thus helping in penetrating the host cells (Moreno et al., 1994; Yakubu et al., 1994; Dorta et al., 1995; Wilkowsky et al., 1996). This glycoprotein also activates the metacyclic trypomastigote protein tyrosine kinase (Favoreto et al., 1998) triggering an increase in intracellular Ca^2+^ ions concentration in the parasite (Favoreto et al., 1998).

The *T. cruzi* infection occurs in two phases, the first phase is the acute phase, and the second phase is popularly known as the chronic phase. The acute phase remains unnoticed and may be characterized by a localized inflammation at the site of parasite entry (Perez-Molina and Molina, 2018a). In the second stage, popularly called the chronic stage (10–20 years after the infection) (Pinazo et al., 2014a), the parasite causes myocarditis by entering the myofibrils of the heart (Perez-Molina and Molina, 2018a). About a quarter of *T. cruzi*-infected individuals develop chronic chagasic cardiomyopathy (CChC), which is the most serious manifestation of this infection (Brener and Gazzinelli, 1997; Albareda et al., 2006; Marin-Neto et al., 2007; Fares et al., 2013). CChC may occur several years after the initial infection (Urbina, 2010), this condition may lead to cardiac arrhythmias, thromboembolism, as well as cardiac arrest (Marin-Neto and Rassi, 2009; Rassi et al., 2009; Urbina, 2010). Unfortunately, despite extensive pre-clinical and clinical investigations on this infection, the pathogenesis of CChC is still unclear and requires more elaborate investigation (Soares et al., 2001; Zacks et al., 2005).

## Current Therapeutic Advancement for Combating Chagas Disease and Their Limitations

Presently for the treatment of *T. cruzi* infection, the only drugs available are Nifurtimox and benznidazole (Croft et al., 2005; Gurtler, 2007). However, the use of Nifurtimox is limited on account of its major side effects like renal and hepatic impairment, along with adverse effects on neurological and gastrointestinal functions, for benznidazole the most accounted side effect is the development of hypersensitivity reaction. Besides the development of resistance against these drugs has also posed a major hindrance in the successful treatment of this disease (Wilkinson et al., 2008; Cattaneo et al., 2010; Campos et al., 2014).

However recent trials of some new drugs (Kaiser et al., 2011) and combination therapy (Cavalli et al., 2010) have shown promising potential in combating the infection. For instance, nifurtimox-eflornithine combination therapy (NECT) (Barrett et al., 2007; Maya et al., 2007; Kansiime et al., 2018) showed promising results for the treatment of cerebral stage of disease in African trypanosomiasis, which were found to show elevated efficacy and reduced therapeutic cost. Some of the other drugs namely 2-piperazine-1-ylquinazoline-4-ylamine derivative and lapachol (Cavalli et al., 2010), UR-9825 and triazoles (Urbina et al., 2000; Urbina, 2015), N-methyl-piperazine-urea-F-h Fvinyl-sulfone-phenyl, and semicarbazone scaffold (Urbina and Docampo, 2003), bisphosphonate (Montalvetti et al., 2001), allopurinol (Berens et al., 1982; Natto et al., 2005), miltefosine (Saraiva et al., 2002) and their corresponding targets namely trypanothione, P-450-dependent C14α-demethylase, squalene synthase (Shang et al., 2014), cruzipain inhibitor, farnesyl pyrophosphate synthase (Montalvetti et al., 2001), purine salvage inhibitors (Berg et al., 2010) and prenyl and N-myristoyl transferase inhibitors (Frearson et al., 2010) respectively have shown promising results against the disease although their extensive *in vitro* and *in vivo* efficacy and clinical studies are yet to be carried out (Table 1).

**TABLE 1:** Drugs with potential trypanocidal activity with their stage of treatment and their mode of transport.

S. No.	Pharmaceutical agents	Treatment stage	Mode of transport	References
1.	Eflornithine	Second stage	Amino acid transporter AAT6	Barrett et al., 2007
2.	Allopurinol	Second stage	nucleobase transporters, H1 and H4	Natto et al., 2005
3.	Nifurtimox	First and second stage	P2 aminopurine transporter	Barrett et al., 2007
4.	Diminazene	First and second stage	TbAT1/P2	Matthews, 2005
5.	Bisphosphonate	First and second stage	Endocytic pathway	Montalvetti et al., 2001
6.	Benznidazole	First stage	Passive absorption	Maya et al., 2007
7.	Suramin	First stage	Endocytic pathway invariant surface glycoprotein ISG75	Gutteridge, 1985
8.	Melarsoprol	Second stage	P2-purine transporter	Barrett et al., 2007

## Nanotechnological Strategies for Combating Chagas Disease

**FIGURE 2:**
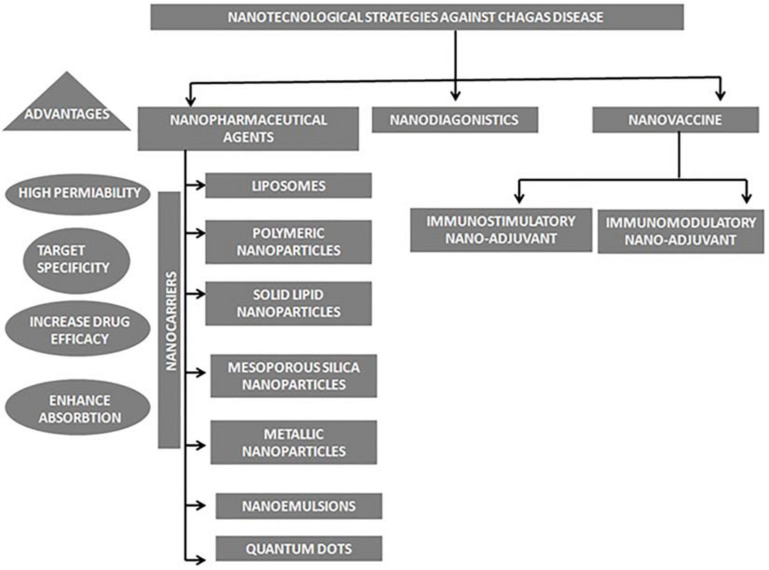
Schematic representation of various nanotechnological strategies used in treatment of Chagas disease.

Nowadays new development in the field of nanoscience has opened new horizons for the treatment of Chagas disease; nano-pharmaceutical agents of various physical and chemical properties are under study which could enhance the efficacy of anti-chagasic drugs to a great extend, moreover the field of nanodiagnostics and nano-vaccine also have provided hope for successful detection and eradication of this infection (Figure 2).

### Nanopharmaceutics

Nanopharmaceutic agents like nanocarriers, nanoemulsions, quantum dots may play a pivotal role in drug delivery against Chagas disease. The function of nanocarriers is largely determined by their structure and physical properties, which can be strategically explored to target these intracellular parasites (Quijia Quezada et al., 2019). The vital factors which determine the performance of nanomaterials are particle size, shape, surface charge, mechanisms, and patterns of bio-distribution (Nel et al., 2009). For the treatment of Chagas disease, the ideal characteristics that these nanomaterials must show are high distribution volume and enhanced circulation time in the body (Nifurtimox or Benznidazole) (Morilla and Romero, 2015). Many reports suggest that anionic nanoparticles depict increased circulation time in blood compared to cationic ones (Arvizo et al., 2011). But, due to the negative nature of cell membranes, neutral nanoparticles, and little negative charge, show significantly longer circulating half-lives (Gratton et al., 2008; Osaka et al., 2009; Serda et al., 2009). Similarly, nanoparticles, with an average size of less than 200 nm, aids in longer circulation time in the bloodstream. A large number of studies have been reported with nanoparticles of various physical and chemical characteristics, that have shown promising roles in enhancing the efficacy of anti-trypanosomal drugs (Ferreira et al., 2016; Quijia Quezada et al., 2019); however, for a majority of these nano pharmaceutical agents, much detailed research is required before their successful use in the clinical platform (Table 2).

**TABLE 2:** List of different nanomaterials with varying composition that have been used for drug delivery in pre-clinical studies of Chagas disease.

No.	Nanomaterial	Composition	Active agent	Preparation method	Size (nm)	ZP (mV)	References
1.	Liposomes	pH-sensitive liposomes	ETZ	Extrusion	379	NI	Morilla et al., 2005a
			Amphotericin B	Not indicated	NI	NI	Cencig et al., 2011
			Stearylamine	hydration			Yoshihara et al., 1987
2.	Polymeric nanoparticles	(a) poly-ε-caprolactone	Urosolic acid	Nanoprecipitation	172.2	36	Abriata et al., 2017
		(b) PLA-PEG	Bis-triazole D0870	Simple emulsification	100–200	NI	Molina et al., 2001
		(c) NC-PCL-PLAPEG	LYC	Nanoprecipitation	105.3	NI	Branquinho et al., 2014
		(d) PCL-PLA-PEG	LYC	Nanoprecipitation	100–250	−30 to -57	Branquinho et al., 2017
		(e) RSNO	Nitric oxide	Ionotropic gelation	270–500	NI	Contreras Lancheros et al., 2018
		(f) SEDDSs	RAV	Self-emulsifying	100–250	−45 to -57	Sposito et al., 2017
		(g) PACA	Nifurtimox	Emulsion polymerization	≤200	NI	Gonzalez-Martin et al., 1998
			Allopurinol				Gonzalez-Martin et al., 2000
		(h) Multiparticulate benzonidazole polymers	BNZ	Nanoprecipitation and freeze-drying	233	35.4	Seremeta et al., 2019
3.	Solid lipid nanoparticles	H_2_bdtc-SLNs	S-benzyldithiocarbazate	High-pressure homogenization and microemulsion	127.4	−56.1	Carneiro et al., 2014
4.	Mesoporous–silica nanoparticles	mesoporous silica nanoparticle and chitosan coating	BNZ	Hydration	3.3	−11.5	Hu et al., 2014
5.	Nanoemulsions	Sulfonamides	Clove oil	Emulsification	35–100	NI	Vermelho et al., 2018
			Ursolic acid		57.3		Vargas De Oliveira et al., 2017
			BNZ		241.6	−71.41	Streck et al., 2019
6.	Quantum dots	–	CdTe	Colloidal chemistry	NI	NI	Vieira et al., 2011

**BNZ, Benznidazole; mv, millivolt; PACA, Poly (alkyl cyanoacrylate) nanoparticles; PN, Nanoparticles with poly-ε-caprolactone; NC, Nanocapsules; PCL, Poly-ε-caprolactone; PLA, PEG- polyethylene glycol-polylactide; ZP, Zeta potential; nm, nanometer; RAV, Ravuconazole; SEDDSs, Self-emulsifying drug delivery systems; NI, no indication.**

#### Liposomes

Liposomes are vesicles like two-dimensional nanostructures, consisting of a lipid bilayer made of phospholipids and cholesterol molecules, and possess separated hydrophilic and hydrophobic portions (Huang, 2008; Li et al., 2019). Previous reports suggest that the encapsulation of drugs in liposomes can significantly alter the pharmacokinetics of the drug. The efficacy of the liposomal formulation is due to its ability to deliver the drug molecule to the target site over a prolonged period and also attenuate the toxic effects of the drug (Allen and Cullis, 2013; Abu Lila and Ishida, 2017). A wide range of molecules such as ribosomes, proteins DNA, immunological adjuvants, or drugs can be delivered using these agents, which aids in increasing the therapeutic efficacy, biocompatibility, and reducing toxicity in biological systems (Samad et al., 2007; Cencig et al., 2011; Mody et al., 2014; Garg et al., 2015; Bulbake et al., 2017).

Many reports suggest that benznidazole (BNZ) loaded in multilamellar liposome vesicles (MLV) depicted much higher accumulation in the mice liver and lower blood BNZ concentrations compared to free BNZ treatment (Yoshihara et al., 1987; Morilla et al., 2005b). Although, this strategy did not affect the parasitic loads’ levels in *T. cruzi* infected mice (Alba Soto et al., 2003). The use of some drugs with very potent anti-trypanocidal activity like hydrogenated trypanocidal etanidazole (ETZ) is limited due to its hydrophilic structure which results in a slow diffusion rate through the membrane. But in comparison to free ETZ, pH-sensitive liposome-encapsulated ETZ (L-ETZ) depicted much efficient anti-amastigote activity *in vitro* and *in vivo* studies (Morilla et al., 2005a). However, due to its pH-sensitive nature and poor penetration capacity in many cells like spleen cells, muscle cells, nerve cells, hepatic macrophages the use of this nanocarrier system is limited. Therefore it can be inferred that further study should be conducted with this nanocarrier, with surface charge modifications that may aid in immune system evasion and elevate its penetration capacity in most cells.

#### Polymeric Nanoparticles

These nanomaterials are made up of solid colloidal particles that can be dissolved, entrapped, encapsulated, or adsorbed onto the constituent polymer matrix. These nanocarriers enhance the bioavailability of the drugs to a great extent (Gonzalez-Martin et al., 2000; Morgen et al., 2012). A large number of natural or synthetic polymers may be used to produce these nanomaterials, such as poly(lactide-co-glycolide) (PLGA); polylactide (PLA); polyglycolide (PGA); polycaprolactone (PCL); poly(D, L-lactide); chitosan, and PLGA-polyethylene glycol (PEG) (Prabhu et al., 2015). The properties that make these polymers a very apt candidate for drug carrier are their biodegradability and biocompatibility (Pinto et al., 2013; Branquinho et al., 2017) and are therefore approved by the U. S. Food and Drug Administration (FDA) (Gonzalez-Martin et al., 1998; Morilla and Romero, 2015; Prabhu et al., 2015; Zhao et al., 2016). A large number of studies with polymeric nanoparticles as nanocarriers have been documented (Table 2). A previous study (Gonzalez-Martin et al., 1998) reports the use of nifurtimox loaded in poly(alkyl cyanoacrylate) nanoparticles (PACA), measuring less than 200 nm, their degradation rate is slower and was found to be less cytotoxic since they were composed of long alkyl chain (Muller et al., 1990). Besides *in vitro* assays using these nanoparticles loaded with nifurtimox showed very efficient anti-epimastigote activity, compared to free nifurtimox (40%. anti-epimastigote activity). Another study showed the use of sterols in polyethylene glycol-polylactide (PEG-PLA) nanospheres (Molina et al., 2001), that had a size between 100 and 200 nm, along with 90% drug encapsulation efficiency for bis-triazole, although this formulation did not show satisfactory *in vivo* efficacy. Nitric oxide plays a pivotal role in the modulation of the immune system against intracellular pathogens (Molina et al., 2001). After infection with *T. cruzi*, many immune cells such as dendritic cells, macrophages (Gil-Jaramillo et al., 2016), show growth inhibitory effects on the pathogen by releasing several effector molecules, like NO (Seabra et al., 2008; Gutierrez et al., 2009). However, as a free radical, NO is unstable in the biological system. The use of low molecular weight S-nitrosothiols (RSNO), has been reported as promising NO donors in many previous reports (Amadeu et al., 2007; Contreras Lancheros et al., 2018) this also increases NO half-life. However, owing to their thermal unsteadiness, the use of RSNO’s is limited. Recently some studies have shown the use of polymer nanoparticles that helps in constant NO release resulting in high anti-trypanosomal activity. Another self-emulsifying drug delivery system (SEDDS that consists of a mixture of drug, oil, surfactant, and occasionally a solvent, it is also a potent drug delivery agent (Kohli et al., 2010). Ravuconazole (RAV), which is an effective anti-*T. cruzi* drug (epimastigote and amastigote) was prepared by SEDDS comprising of isotropic mixtures of anhydrous oil and surfactant (Sposito et al., 2017). This formulation depicted elevated anti-amastigote activity in comparison to the drug alone in *in vitro* assays. This formulation was also found to have very insignificant toxicity in *in vivo* studies thus making it a potent candidate for pre-clinical anti-trypanosomal studies. Lychnopholide (LYC) is another compound of herbal origin, which have depicted very potent anti-trypanosomal activity in many assays, but owing to its low solubility and high lipophilicity, its use is restricted. Thus, LYC-loaded nanocapsules (NC) were prepared using poly-ε-caprolactone (PCL) and poly (lactic acid) polyethylene glycol (PLA-PEG) polymers, these nanocapsules depicted much higher drug efficacy in *in vivo* studies (Branquinho et al., 2014), which is due to the nanocarrier’s ability to increase the drug release period in biological media. Another study reported the utilization of ursolic acid-loaded in nanoparticles comprising of poly-ε-caprolactone (PN-UA) (Abriata et al., 2017) as a potent formulation against Chagas disease, however, it’s use was limited due to it’s low bioavailability. Thus, it can be said that a large number of these polymeric nanomaterials may be a potent candidate for anti-chagastic drug delivery agents and may be very useful in future clinical trials for Chagas disease.

#### Solid Lipid Nanoparticles (SLN)

These nanoparticles are composed of a mixture of solid and liquid lipid, in which the lipid matrix is solid at room and body temperature. These are quite stable and can encapsulate drugs with controlled release. Moreover, they have good biocompatibility, biodegradability, high bioavailability, and also can be produced on a large scale (Muller et al., 2000; Mehnert and Mader, 2001; Wissing et al., 2004). SLN have shown promising results as oral and percutaneous drug delivery agent, by convalescing the solubility and dissolution rate of the drugs, elevating their contact with the epithelium and attenuating their efflux by the cellular drug transporters (Scioli Montoto et al., 2020). Moreover, 5-Hydroxy-3-methyl-5-phenyl-pyrazoline-1- (S-benzyldithiocarbazate) (H2bdtc), is another compound that has been reported to be effective against Chagas disease but owing to its low solubility, its use is limited. Thereafter in one study, H2bdtc-SLN was synthesized (Muller et al., 2000; Carneiro et al., 2014) and this was found to be very effective at concentrations significantly lower than those normally used for benznidazole. Besides, *in vivo* studies reported that this formulation attenuated inflammation and damage to the heart and liver in *T. cruzi* infected mice. Moreover, this formulation was observed to be much effectual on the parasite than the drug alone. Immuno-stimulant complexes (ISCOM) (Morein et al., 1984), are lipids comprising of Quillaja Saponaria of homogeneous size (40 nm) that are used as adjuvants for vaccines (Wilson et al., 2014), namely antigen carriers, DNA vaccines for administration in oral and intranasal route (Lendemans et al., 2007; Sun et al., 2009). ISCOM encapsulated actinomycin D with antibodies resulted in dose-dependent effects (Cruz-Bustos et al., 2012) and were found to be more effective compared to antibiotic alone, indicating this formulation as a very potent nanocarrier for anti-trypanosomal therapy.

#### Mesoporous Silica Nanoparticles

Currently, a study has been reported with, solid inorganic material, mesoporous silica nanoparticles and chitosan coating, that depicted very efficient anchoring with benznidazole molecules (Hu et al., 2014). These nanoparticles are characterized by a complex network of well-organized pores of homogeneous sizes that helps in the functionalization of the antichagasic drugs or any other components. These nanoparticles were found to have uniform shapes and sizes over 3 nm, with a zeta potential around -11 mV. Besides, these particles were found to extremely high anti-epimastigote activity compared to free BZ in *in vitro* assays (Quijia Quezada et al., 2019), indicating these particles as a very potent nano-carrier for drug delivery in anti-chagasic studies.

#### Metallic Nanoparticles

These nanoplatforms are composed of metal atoms clusters, whose size shape, and structures decide their optoelectronic and physicochemical characteristics. These nanoparticles are widely used for many applications, but their use is sometimes limited due to their potential toxicity. One confocal microscopic study reported the intracellular endocytosis of gold nanoparticles marked with transferrin in epimastigotes (Eger and Soares, 2012) which showed two specific regions of the pathogen, namely the lower portion of the cytopharynx and reservosomes, that can be visualized using this technique. This assay depicted a promising method for immunolocalization study with gold nanoparticles labeled with secondary antibodies in the pathogen (Quijia Quezada et al., 2019).

#### Nanoemulsions

Nanoemulsions have shown promising potential as apt drug delivery agents. These particles are thermodynamically stable isotropic system comprising of two immiscible liquids that are mixed in a single phase with the aid of an emulsifying agent (Tomiotto-Pellissier et al., 2017). The size of these nanoparticles may vary 10 to 600 nm (de Araujo et al., 2007; Tomiotto-Pellissier et al., 2017), that are composed of a mixture of insoluble liquids in two separate phases where the vesicles that are in a dispersed phase are bordered by a continuous phase, hence making them very potent nanocarriers for increasing the solubility of hydrophobic drugs. A wide variety of surfactants are used for the stabilization of the emulsion (Sarker, 2005). A study was reported with an anti-trypanosomal molecule, ursolic acid in nanoemulsions, which was found to have high stability with a size of 57.3 nm (Vargas De Oliveira et al., 2017). This formulation was found to have excellent anti-amastigote activity and was found to be non-toxic in fibroblast cells in -vitro. Another study reports a nanoemulsion formulation formed with clove oil in sulfonamide, which was observed to possess carbonic anhydrase inhibitor activity which targets the α-class enzyme from *T. cruzi* and was found to have excellent anti-trypanocidal activity (Vermelho et al., 2018). Another nanosystem was recently formulated that releases benznidazole from oil nanoemulsions (Streck et al., 2019). They were characterized with a diameter around 240 nm, and zeta potential around -71 mV, and had physicochemical properties perfect for enhancing water solubility of drugs, with a slow drug release rate. This formulation was found to be non-toxic in Vero cells with excellent anti-trypanocidal activity. very little cytotoxicity in Vero cells with excellent anti-trypanocidal activity. From these results, it can be inferred that nanoemulsions can be a very potent drug delivery system for anti-chagasic studies.

#### Quantum Dots

Quantum dots (QDs) nanoscale crystal structures that can transport electrons. When UV light illuminates these semiconducting nanoparticles, they can emit lights of various colors, this fluorescence properties make them an ideal nanomaterial for a large range of applications in biomedicine for drug delivery and diagnosis (Ho et al., 2013; Quijia Quezada et al., 2019). But, these nanoparticles depicts adverse side-effects *in vitro* and *in vivo* assays (Hardman, 2006), hence posing potential risks to human health. In one study the effect of quantum dots of cadmium tellurium (CdTe) was investigated on *T. cruzi* epimastigotes (Vieira et al., 2011), which revealed that high doses of this nanomaterial led to a steady decline in *T. cruzi* growth patterns, encouraging further investigation in this field. Besides significant morphological alterations in *T. cruzi*, such as DNA damage, the formation of blisters in the plasma membrane, and mitochondrial swelling were observed when incubated with 200 μM CdTeQD. However, when incubated with a low concentration of CdTe QDs (2 μM) no significant alterations were observed in the pathogen, moreover, their proliferation rate remained unaltered. Thus it can be said that although a lower concentration of CdTe QDs (2 μM) may be quite useful for bio-imaging, its use at a higher concentration is limited due to its toxicity (de Araujo et al., 2015).

### Nano-Diagnostics

Chagas disease can be diagnosed by two methods namely parasitological and serological (Nagarkatti et al., 2014), based on the stage of infection (Morilla and Romero, 2015), moreover, these phases of infection have been reported to express a large number of biomarkers (Pinazo et al., 2014b; Pinho et al., 2016) (Table 3), that if aptly detected could lead to accurate detection of the disease. The field of nano-bio sensors has opened a new horizon for efficient detection of trypanosomiasis with greater accuracy and sensitivity (Sousa et al., 2014). A well-known non-invasive congenital Chagas disease detection system known as Chagas urine nanoparticle assay (Chunap) is an extremely effective and sensitive detection technique that can and detect the antigens from the patient’s urine (Castro-Sesquen et al., 2014). This assay has the ability to identify *T. cruzi* antigens from different chemical structures using polyunsaturated N-isopropyl acrylamide and trypan blue functionalized nanoparticles.

**TABLE 3:** List of diagnostic tests, biomarkers, and challenges for diagnosis at different phases of Chagas disease.

Disease phase	Diagnostic test	Challenges	Biomarkers	References
Acute phase	Serological test: blood wet smear, blood concentration technique such as microhematocrit or Strout technique	Symptoms are non-specific or absent.	IL-12, TNFα, IFNγ, NO, IL-17, IL-10, CD4 + Tcells	Andrade et al., 2014
Chronic phase	Parasitological test: Detection of anti *T. cruzi* antibodies by conventional or recombinant ELISA, indirect hemagglutination assay. Indirect immunofluorescence assay or immunochromatographic assay and PCR (parasitologic)	Cross-reaction with antibodies from patients infected with *Leishmania sp*. or *T. rangeli*	Aptamer, CCL2, MAL/TIRAP, CCR5, CD15s + Treg cells, CD27 + T cells, CD28 + T cells, CD8 + T cells, TIMP-1, IMP-2, Troponin I, TGF-β, IL-10, APOA1, Fibronectin, MMP-2, MMP-9, ANP, BNP, N-terminal pro-BNP, IFN-γ, TNF-α, IL-1β, IL-6, CKMB, miRNA-1, miRNA-133a, iRNA-133b, miRNA-208a, miRNA-208b, PIIINP, PICP, Syndecan-4, ICAM-1, Galectin-3, KMP11, HSP70, PAR2, Tgp63, Antigen 13, SAPA, Tc24	Castro-Sesquen et al., 2013; Fares et al., 2013; Pinto et al., 2013; Ferreira et al., 2014; Nagarkatti et al., 2014; Pinazo et al.,2014a,b; Santamaria et al., 2014; Sousa et al., 2014; de Araujo et al., 2015; Keating et al., 2015; Miyara et al., 2015; Requena-Mendez et al., 2015; Pinho et al., 2016

Apart from this, another efficient nano-diagnostic biosensor systems are nanowires (NW), that are much more competent in comparison to their thin film analogs (Patolsky et al., 2006; Keating et al., 2015). The competency of the transistors of field effect transistors (nano-FET) used in various biosensors was enhanced extensively by using biomarkers immobilized with biocompatible ethanolamine and polyethylene glycol derivatives (Janissen et al., 2017). Moreover, this technique depicted a major improvement in specificity and capture efficiency of target biomolecules for the pathogenic antigens (Castro-Sesquen et al., 2013), with extreme sensitivity (Quijia Quezada et al., 2019). Therefore it can be said that this nanowire biosensor system provided us an extremely sensitive tool for the detection of the infection, thus aiding in better disease diagnosis.

The electrochemical immune-sensing device called Nanopoc^®^ (Aydin et al., 2019) is another significant advancement in field of diagonosis. This device is composed of four recombinant proteins immobilized on silica-coated super-paramagnetic iron oxide nanoparticles. In this assay, after formation of the antigen-antibody complex in the solution, these nanoparticles are attracted toward the electrode with the aid of a magnetic field, here the result is measured by electrochemical sensing. This strategy was found to be more efficient, sensitive and less time consuming compared to direct electrode immobilization method (Morilla and Romero, 2015). Moreover very recently, a micro fluidic immunosensor was produced utilizing a carbon electrode, which was modified by electro-deposition of gold nanoparticles and functionalized with proteins of *T. cruzi* epimastigote form; this device helped in efficient quantification of anti*-T. cruzi* antibodies from serum samples (Pereira et al., 2011). Quantification of antibodies in serum samples can also be done using another nano-biosensor that utilizes horseradish peroxidase enzyme-labelled secondary antibodies against human IgG, along with 4-tert-butyl catechol (4-TBC) that acts as an enzyme mediator (Morilla and Romero, 2015). Thus it can be inferred that accurate usage of these nano-bio sensors could play a pivotal role in apt detection of the infection and thus aid in controlling this endemic infection.

### Nanovaccines

A wide range of therapeutic vaccines against Chagas disease have been tested in animal models, which ranges from using the whole parasites, or purified and recombinant proteins, to viral vectors and DNA vaccines (Quijano-Hernandez and Dumonteil, 2011; Vazquez-Chagoyan et al., 2011; de Araujo et al., 2015). But owing to reduced immunogenicity, the use of recombinant antigens are limited. Furthermore apart from the alum-containing vaccines, no other vaccines were approved by FDA up till 2009, when 3-*O*-desacyl-4’- monophosphoryl lipid A was approved. However the efficiency of the adjuvants like alum and monophosphoryl lipid A are limited due to their poor ability to induce a T-cell immune response (Morilla and Romero, 2015). Here nanoparticulate antigen delivery systems can aid the uptake of antigens by antigen-presenting cells thereby serving as a measure for controlled release of antigen (Rodriguez-Morales et al., 2013).

A previous study has been reported whereby a single nanostructure was developed using (ISPA) immunostimulatory nanoparticles (Bertona et al., 2017), that was around 42 nm in size with a zeta potential of -1.96 mV. These nanomaterials showed a much efficient immune response against the pathogen when compared with several adjuvants such as Freund Adjuvant (FA), aluminum hydroxide (AH) in female BALB/c mice. Besides, *in vivo* immunization assay in animal models depicted complete protection against the pathogen when immunized with ISPA and trans-sialidase (TS). Additionally, the liposomes carrying reconstituted membrane proteins of the parasite have proved to be a useful agent for immunization in many studies (Mesa et al., 2004; Kita et al., 2005).

Tc24 a protein found in the flagellar region of *T. cruzi* has long been known as a potent antigen target (Krautz et al., 2000). Immunization with this antigen was found to create major protective activity against *T. cruzi* infection (Taibi et al., 1993, 1995). Another study reports the use of a PLGA (poly (lactic-co-glycolic acid)) nanoparticle-based vaccine composed of oligodeoxynucleotides having the CpGm motif (CpG ODN) as an immunomodulatory adjuvant, that depicted a very potent immune response (Vabulas et al., 2000; Speiser et al., 2005). PLGA nanoparticles formed along with CpG ODN and recombinant Tc24 antigen depicted an effect very similar to Aluminum hydroxide Gel (Alhydrogel^®^) in *in vivo* assays. The PLGA nanostructure aided in prolonging Tc24 antigen release. Moreover, this vaccine produced a rapid T-cell response. This report indicated the PLGA nanoparticles as a potent nanocarrier for hapten delivery that may elevate the efficacy as well as immunogenecity of a protein-based vaccine for Chagas disease (Barry et al., 2016). The desirable features of a vaccine against *T. cruzi* infection are their ability to attenuate the parasitic burden, tissue inflammation, as well as prevention of cardiac tissue damage, thereby causing an increase in host survival (Rocha et al., 2003; Hyland et al., 2007). Thus here it is extremely evident that nanostructural systems or immunogenic vehicles are much more efficient than current standard therapies (Araujo et al., 2005; de Alencar et al., 2007; Migliaccio et al., 2008; Miyara et al., 2015).

## Discussion

The nanotechnological strategies for drug and vaccine administration with the aid of nanocarriers embody a very potent strategy for Chagas disease treatment. This study made it very evident that a wide variety of nano-pharmaceutical drug delivery agents and diagnostic apparatus could open a new horizon of medical technology for the successful treatment of Chagas disease. However, the pre-clinical uses of these nano-medical technologies are encountered with some major challenges namely economic burden, especially for the developing countries, the elevated healthcare costs for this infectious disease produce an unfavorable cost-benefit ratio (Wilson et al., 2005). Besides, in-adequate pre-clinical data in favor of these nanotechnological interventions also accounts as a major disadvantage against these nanomedical strategies (Morilla and Romero, 2015). Thus this situation demands a concerted approach from governmental and private agencies in conjunction with pharmaceutical industries focusing on the efficient implementation of these nanomedical strategies that will elevate the efficacy of anti-chagasic treatment procedures.

## Conclusion

The danger of Chagas disease has long been unnoticed by both government and private pharmaceutical industries which hindered the pathway of new drug discovery and novel efficient treatment strategies against this infection. This zoonotic tropical infection has adversely affected both human and farm animal’s health in endemic regions and also poses a great danger for the near future if not controlled efficiently. The present review has shed light on the present treatments used against Chagas disease along with their limitations. It has majorly focused on the implication of nanotechnological interventions for enhancement of existing therapeutic options available for the treatment of Chagas disease. The nanocarriers encapsulated anti-trypanocidal drugs has shown enhancement of trypanocidal activity in both vitro and *in vivo* studies, besides, these nanostructures have also increased the efficacy of the drugs. However, limited attempts have been made to explore the potential of these potent nanocarriers in pre-clinical studies. The use of nano-bio sensors, where nanodevice supplemented electrochemical immunoassay has also opened up new horizons for accurate and efficient detection of this infection at very early stages. Moreover, the use of nanomaterials to increase the immunogenicity of anti-chagasic vaccines can be very useful soon for the successful eradication of this infection. Although the use of nanotherapeutics against Chagas disease is in very nascent stages, appropriate study and application of these strategies against Chagas disease may play a pivotal role in the eradication of this infection.

## Author Contributions

SC designed and wrote the complete manuscript.

## Conflict of Interest

The author declares that the research was conducted in the absence of any commercial or financial relationships that could be construed as a potential conflict of interest.
